# Splenectomy Normalizes Hematocrit in Murine Polycythemia Vera

**DOI:** 10.1371/journal.pone.0007286

**Published:** 2009-09-30

**Authors:** Jan-Rung Mo, Anjili Mathur, Minilik Angagaw, Shuxia Zhao, Yuxun Wang, Diana Gargano, Alessandra DiBacco, Eric S. Bachman

**Affiliations:** Departments of Oncology-Pharmacology, Lab Animal Research and Pharmacology Merck Research Laboratories, Boston, Massachusetts, United States of America; INSERM U567, Institut Cochin, France

## Abstract

Splenic enlargement (splenomegaly) develops in numerous disease states, although a specific pathogenic role for the spleen has rarely been described. In polycythemia vera (PV), an activating mutation in Janus kinase 2 (JAK2^V617^) induces splenomegaly and an increase in hematocrit. Splenectomy is sparingly performed in patients with PV, however, due to surgical complications. Thus, the role of the spleen in the pathogenesis of human PV remains unknown. We specifically tested the role of the spleen in the pathogenesis of PV by performing either sham (SH) or splenectomy (SPL) surgeries in a murine model of JAK2^V617F^-driven PV. Compared to SH-operated mice, which rapidly develop high hematocrits after JAK2^V617F^ transplantation, SPL mice completely fail to develop this phenotype. Disease burden (JAK2^V617^) is equivalent in the bone marrow of SH and SPL mice, however, and both groups develop fibrosis and osteosclerosis. If SPL is performed after PV is established, hematocrit rapidly declines to normal even though myelofibrosis and osteosclerosis again develop independently in the bone marrow. In contrast, SPL only blunts hematocrit elevation in secondary, erythropoietin-induced polycythemia. We conclude that the spleen is required for an elevated hematocrit in murine, JAK2^V617F^-driven PV, and propose that this phenotype of PV may require a specific interaction between mutant cells and the spleen.

## Introduction

The mammalian spleen, an organ that is not required for adult human life, has rarely been proposed to play a specific role in human disease. That is, despite pathologic enlargement (splenomegaly) in numerous conditions, splenectomy rarely results in reversal of the underlying disease process. Splenomegaly often reflects an expansion of cellular proliferation outside of the bone marrow compartment, and is called extramedullary hematopoiesis (EMH). This process can occur not only in spleen, but also in liver, kidney and lymph nodes. Whether EMH plays a required or permissive role in myeloproliferative disorders (MPDs) is unknown. Splenomegaly is observed in patients with the Philadelphia chromosome positive MPD chronic myelogenous leukemia (CML), for example, but splenectomy fails to affect disease progression [1], [2]. On the other hand, the role of the spleen in the progression of another MPD, polycythemia vera (PV), has not been tested due to frequent surgical complications [3]–[5]. The pathologic hallmark of PV is increased red blood cell mass (hematocrit) that is not mediated by the hormone erythropoietin (Epo). Thus, PV can be distinguished from secondary polycythemia by cell-autonomous proliferation or red blood cells.

In 2005, an activating mutation in the nonreceptor tyrosine kinase Janus kinase 2 (JAK2^V617F^) was found to be present in the blood in more than 90% of patients with human PV [6]–[10]. Moreover, introduction of JAK2^V617F^ into hematopoietic cells causes PV in mice, and inhibitors of Jak2 (Jak2i's) reduce hematocrit and spleen size [11]. We also observed that the efficacy of Jak2i's in such JAK2^V617F^-driven PV in mice is disproportionately associated with reduced disease burden entirely in spleen, with minimal effect in bone marrow (data not shown). Thus, efficacious treatment of murine PV with Jak2i's suggests that the spleen plays a critically important role in PV. In order to specifically test the role of the spleen in JAK2^V617F^ –driven, murine PV, therefore, we performed surgery either prior to or following polycythemia-inducing bone marrow transplant of JAK2^V617F^ cells, and characterized the resulting phenotype.

## Methods

### Animals

Six-week-old C57Bl/6 (B6) or Balb/c donor mice were obtained from Taconic Farms Inc. (Germantown, NY). The mice were maintained 5 to a cage in pathogen-free conditions. All animal studies were conducted in accordance with protocols approved by the Institutional Animal Care and Use Committee (IACUC).

### Statistical Methods

A Student's t-Test, assuming equal variance, was used to compare hematology parameters between sham (SH) and splenectomized (SPL) mice.

### Expression vectors and viral supernatants

The murine Jak2 cDNA was cloned into the retroviral vector MSCV-IRES EGFP which was generously provided by Dr. Gilliland, as described [12]. We cultured 293T cells in Dulbecco modified Eagle medium (DMEM) with 10% fetal bovine serum (FBS). Transient cotransfection of 293T cells by equal amounts of expression vectors and packaging plasmid (pCL-Eco, Imgenex, San Diego, CA) and generation of retroviral supernatant were performed using FuGENE (Roche, Nutley, NJ) according to the manufacturer's protocol. Viral supernatant was harvested after 48 hours and was used to transduce bone marrow or NIH 3T3 cells to assess viral titer.

### Jak2 V617F allele burden QPCR

Genomic DNA from 30 ul of mouse peripheral blood or 10 mg of mouse tissue was purified by Qiagen tissue and blood DNA kit (cat #: 69506); 10 ng of genomic DNA was used for 10 ul QPCR reactions in 384-well plate format using ABI 7900HT Real-Time PCR system. Primers: Jak2cDNAF2: CACATAGGAACTATTCAGAGTCTTTC; Jak2MutR3: AGAATGTTCTCCTCTCCACAGTA (additional mutations were added to the primer to prevent amplification of unmatching sequences).

### Bone marrow transplantation and splenectomy surgery of mice

Murine BM transplantation experiments were performed as previously described (paper 1). Briefly, the viral titer was determined by transducing 3T3 cells with supernatant (plus polybrene, 10 µg/mL) and analyzed for the percentage of green fluorescent protein–positive (GFP^+^) cells by flow cytometry 2 days after transduction. C57Bl/6 or Balb/c donor mice were treated for 5 days with 5-fluorouracil (150 mg/kg, intraperitoneal injection). Bone marrow cells from donor mice were harvested by flushing femurs and tibias and were cultured for 24 hours in transplant medium (RPMI+10% FBS+6 ng/mL IL-3, 10 ng/mL IL-6, and 10 ng/mL stem cell factor). Cells were treated by spin infection with retroviral supernatants (1 mL supernatant per 4×10^6^ cells, plus polybrene, plus HEPES 30 µL/4 mL) and centrifuged at 2500 *g* for 90 minutes at 30°C 24 hours before and on the day of transplantation. Whole BM cells (1.0×10^6^) were resuspended in 200 uL Hanks balanced salt solution and were injected into lateral tail veins of lethally irradiated (2×5.5 Gy [550 rads]) C57Bl/6 or (2×4.5 Gy [450 rads]) Balb/c recipient mice.

Mice were anesthetized with isoflurane inhalation. After anesthesia, mice were surgically prepared by first shaving the incision site, followed by preparing the incision site with either Betadine/alcohol scrubs or chlorhexidene scrubs. A small incision was made in the left subcostal abdominal wall. The spleen will be exteriorized through the incision, and a splenectomy will be performed by placing ligatures around the splenic vasculature and removing the spleen. The incision will be closed in two layers using surgical suture material and wound clips. Mice were monitored for recovery from anesthesia and kept until wakeup at 37°C. Control group underwent a sham surgery (SH) and they were maintained in the same conditions.

### Analysis of disease in mice

Peripheral blood was collected from the retro orbital cavity using EDTA glass capillary tubes and analyzed by using the Advia 120 Hematology System (Bayer Diagnostics) according to the manufacturer's instructions.

### Antibody staining and flow cytometry

Isolated bone marrow flushing by the femurs or spleen was strained through a 70-µm strainer in the presence of RPMI and 10% fetal bovine serum. Cells were blocked with Fc-block (BD Biosciences) for 15 minutes on ice, and stained with antibodies in Stain Buffer (BD Biosciences) for 30 minutes on ice. Antibodies used were allophycocyanin-alexa fluor 750 anti-mouse Ter-119 (eBioscience, San Diego, CA) and phycoerythrin anti-mouse CD71 (BD Biosciences). After washing, cells were resuspended in Stain Buffer containing Topo-3 (Invitrogen) to allow discrimination of nonviable cells. Flow cytometry was performed on a FACSLSRII cytometer (BD Biosciences, San Jose, CA). At least 10 000 events were acquired, and data were analyzed using FlowJo software (Tree Star, Stanford University, Stanford, CA). Results are presented as dot plots of viable cells selected on the basis of scatter and Topo-3 staining. For erythroblast analysis as previously described [13].

## Results

**Figure 1:**
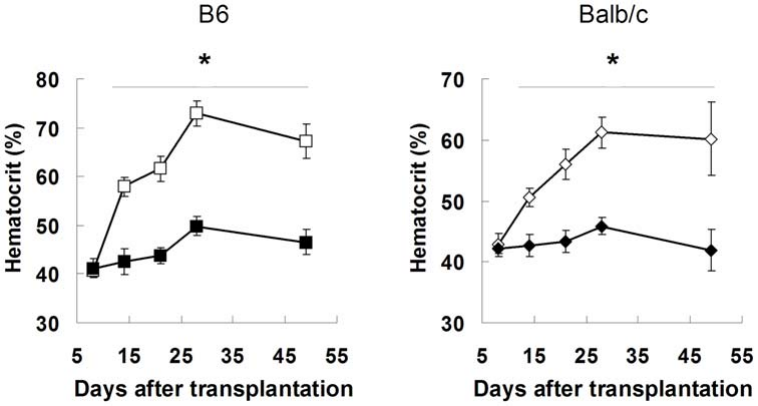
Splenectomy prevents Jak2^V617F^ -driven polycythemia in mice. (A) 6 week old female C57Bl/6 mice underwent splenectomy (SPL, ▪) or sham (SH, □) operations. (B) 6 week old female Balb/c mice underwent splenectomy (SPL, ♦) or sham (SH, ◊) operations. Followed by 2 week recovery, irradiation and injection of Jak2^V617F^ -transduced bone marrow cells from congenic donors. Hematocrit was measured weekly until disease was maximal in sham-operated mice (n = 12/group, error bars indicate SD). Open symbols = sham operated; filled symbols = splenectomy. * indicates p<0.05 compared to SH operated mice.

A well-established mouse model that closely phenocopies human PV was used to test the role of the spleen in PV. Briefly, adoptive transfer of retro virally transduced bone marrow cells containing mutant JAK2^V617F^-GFP into lethally irradiated mice leads to a disease that closely resembles human PV, including elevated hematocrit and splenomegaly [12], [14]. In order to test the role of the spleen in V617F-driven PV, therefore, we removed the spleen (SPL) or performed a sham operation (SH) 2 weeks prior to a bone marrow transplant (BMT) of JAK2^V617F^-containing bone marrow cells. Two strains of mice, C57Bl6 and Balb/c, were used in order to obtain generalizable data for mouse models that differ slightly in disease phenotype [12]. All mice tolerated surgery and the BMT procedure without complication. While SH operated mice rapidly develop PV (Hct = 61.3±3.6 in Balb/c and 73±2.6% in B6 mice), SPL operated mice fail to develop PV over the 7 week observation period (45.8±1.4 and 49.8±1.9%, respectively; Fig. 1). As would be expected after removing the splenic depot of red blood cells (RBCs), RBC mass was higher in SH operated than SPL mice, although this did not reach statistical significance (sTab1). Reductions in other amplified hematopoietic lineages such as white blood cells (WBCs) and platelets were also observed in the SPL cohort, although these were clearly not normalized (sTab1).

**Figure 2:**
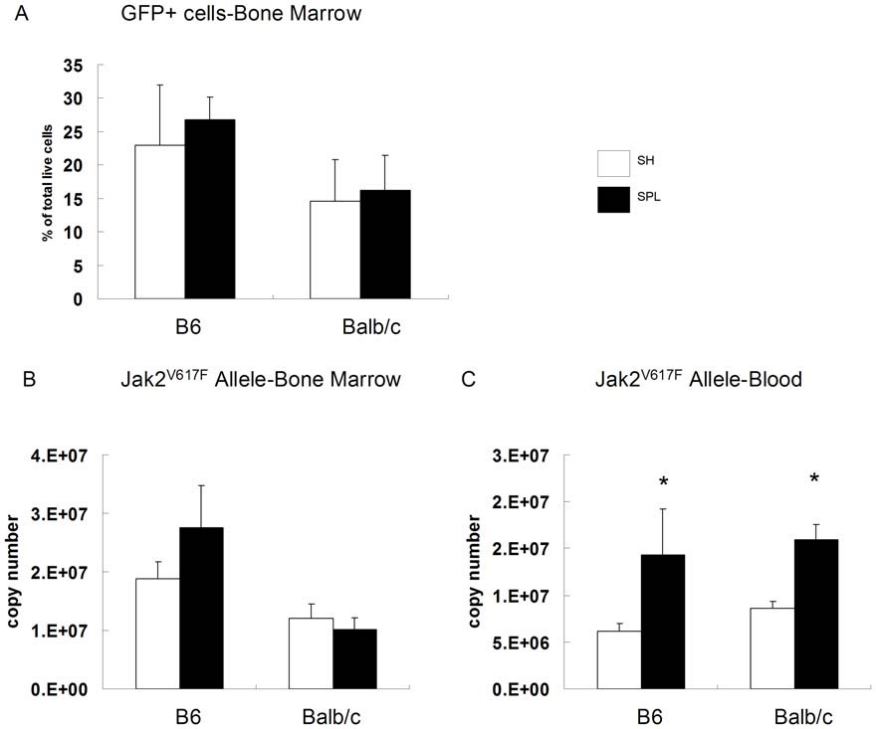
Splenectomy does not alter Jak2^V617F^ disease burden in bone marrow. At the termination of the SPL-BMT study shown in Fig 1, bone marrow cells were harvested and live, GFP+ cells were analyzed by flow cytometry (A) DNA from bone marrow aspirates (B) and blood (C) was also prepared and PCR for Jak2^V617F^ allele was performed. *p<0.01 compared to SH operated mice (n = 6 mice/group in all groups).

In order to rule out the possibility that a lack of PV is due to BMT engraftment failure, we measured JAK2^V617F^-GFP^+^ cells in bone marrow. As shown in Figure 2A, both SH and SPL mice harbor similar, large (15–25%) populations of JAK2^V617F^-GFP^+^ cells. Similarly, mutant JAK2^V617F^ DNA in the bone marrow is readily detectable and similar in SH versus SPL mice (Fig 2B). Surprisingly, mutant JAK2^V617F^ allele burden in the blood of splenectomized mice is more than double the burden in SH (Fig 2C). This implies that the total burden of JAK2^V617F^ disease is comparable in SH and SPL mice because the diseased spleen, approximately 600–800 mg, primarily harbors mutant erythroblasts (sTab1). Lower hematocrits in SPL mice most likely reflects underproduction of RBCs, as opposed to increased turnover, as indicated by normal peripheral blood smears, LDH and bilirubin levels, and no evidence of hemosiderin in macrophages (data not shown). Finally, evidence of extramedullary hematopoiesis is lacking in SPL mice, as indicated by normal liver weights, equivalently low liver erythroid progenitor cells (sTab1), and no pathologic enlargement of lymph nodes by visual inspection. Thus, while JAK2^V617F^ mutation-bearing cells readily engraft into the bone marrows of recipient mice, the lack of a spleen severely impairs or prevents PV.

**Figure 3:**
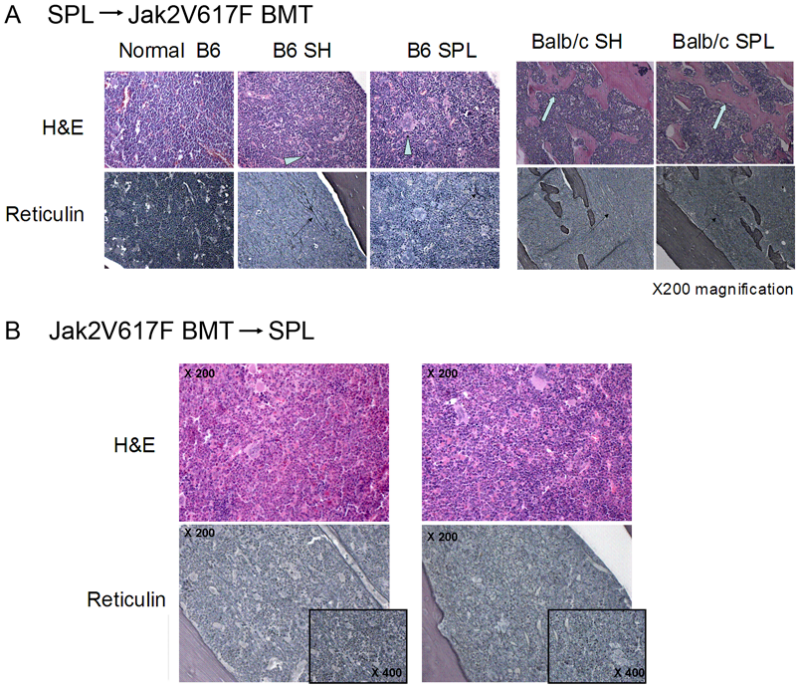
Pathologic fibrosis and osteosclerosis are equivalent in polycythemic and splenectomized, normocythemic mice. A. In the SPL→BMT experiment, histology was performed on tissues from mice approximately 9–10 weeks after SH or SPL. Bone marrow histology shows increased cellularity, prominent megakaryocytes (arrowheads) and reticulin fibrosis (thin arrows in lower panels) in both SH and SPL groups of mice compared to age-matched normal bone marrow. In Balb/c mice, extensive new bone formation (osteosclerosis, aqua arrows) and reticulin fibrosis are found equally in both SH and SPL mice 6 weeks after transplant. B. Splenectomy after V617F-driven PV occurs results in similar hypercellularity (top H & E staining) and reticulin fibrosis in SH and SPL operated mice.

Histological analysis further confirms active JAK2^V617F^-driven disease in both SH and SPL mice (Fig 3). Typically, mice which receive the JAK2^V617F^ BMT sequentially develop myeloid and erythroid hypercellularity, increased numbers of megakaryocytes and reticulin fibrosis. As shown in Fig 3, compared to age-matched, normal B6 mice, megakaryocytosis and reticulin fibrosis is readily identifiable in the bone marrow of both SH and SPL JAK2^V617F^- transplanted mice. Progressive JAK2^V617F^-driven disease in Balb/c mice, as exemplified by new bone formation (osteosclerosis) and extensive, nonlinear reticulin fibrosis is similarly seen in the bone marrow of both SH and SPL mice (Fig 3, right panels). Similarly, mice which undergo SPL after BMT still show hypercellularity and reticulin fibrosis despite normalized hematocrit (Fig 3, lower panels). These findings demonstrate that JAK2^V617F^-driven, elevated hematocrit (polycythemia) can be pathologically independent of JAK2^V617F^-driven fibrosis/osteosclerosis in bone marrow. This would suggest that splenectomy, while effective at reducing JAK2^V617F^-driven pathology in blood (elevated hematocrit), does not alter the pathologic course of JAK2^V617F^-driven PV in bone marrow.

**Figure 4:**
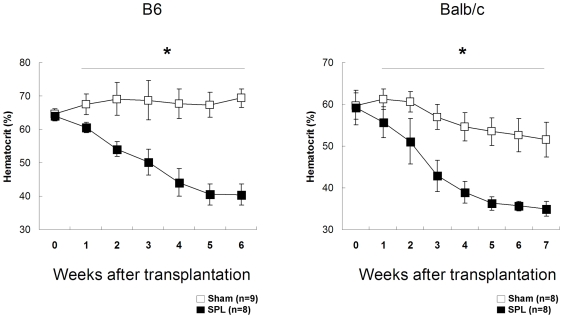
Splenectomy normalizes hematocrit in Jak2^V617F^ -driven polycythemia vera. B6 (left) and Balb/c (right) mice were injected with V617F-transduced, congenic bone marrow cells and allowed to develop polycythemia. After sham (SH, □) or splenectomy (SPL, ▪) operations, mice were followed weekly and hematocrit measured by spun hematocrit. * p<0.05 compared to SH operated mice.

If an intact spleen is required for establishment of PV, is it required for maintenance of PV? In order to test this, PV burdened mice were generated via JAK2^V617F^-GFP BMT and the spleens were removed posttransplant 4 weeks later. In order to assure that PV was at equilibrium, mice were not operated on until hematocrits had uniformly reached maximal, stable values (>64%). As shown in Fig. 4, SH operation has no effect on hematocrit in polycythemic B6 mice, which maintain high hematocrit levels (final 67.4±3.2%). In contrast, SPL results in a rapid decline in hematocrit to normal levels in B6 mice where they are maintained (40.6±3.7%, p<10^−10^). Normalization of hematocrit was observed in 3 separate cohorts of B6 mice at similar rates of descent (data not shown). Balb/c mice responded similarly, although SH operated mice also demonstrated a slight lowering of hematocrit (Fig 4, right). Similar to what is observed when SPL precedes BMT; mutant, GFP+ cells are easily measured in the bone marrow of splenectomized mice (sTab2). Moreover, and as has been well described in prior reports, splenectomy has no effect on hematocrit in normal, non polycythemic mice (data not shown). In conclusion, an intact spleen is required for maintenance of JAK2^V617F^-driven PV. We postulate that the spleen provides either a required niche or a required factor(s) for JAK2^V617F^-expressing hematopoietic cells to maintain the high hematocrit that characterizes PV.

Is a spleen required for polycythemia generally? Polycythemia can result either from a hormone-independent, JAK2^V617F^-driven primary process, or a hormone (Epo)-dependent secondary process. It is clear from prior reports that the spleen is not required for maintenance of hematocrit, nor for regeneration of all hematopoietic lineages following bone marrow transplantation [15]. More specifically, splenectomy in CD1 mice only blunts, but does not prevent secondary, Epo-induced polycythemia [16]. This presents a paradox, because cell autonomous, JAK2^V617F^-driven “true” PV requires a spleen, but cell non-autonomous, or secondary polycythemia may not. Thus, erythropoiesis driven by JAK2^V617F^ results in polycythemia that requires a spleen, while erythropoiesis driven by wild type JAK2 does not.

**Figure 5:**
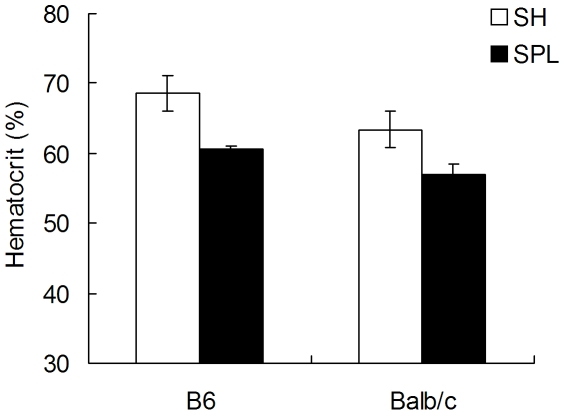
Secondary (Epo-stimulated) polycythemia develops in SH and SPL mice. Eight week old B6 and Balb/c mice (n = 4/group) underwent SH or SPL operations, and 2 weeks later erythropoietin was administered for 7 days. Both SH and SPL mice develop significant polycythemia.

In order to investigate this more fully, we tested the effects of splenectomy on Epo-induced, secondary polycythemia in 2 strains of mice. We first optimized a dosing regimen of recombinant human erythropoietin (Aranesp™) that induces polycythemia in mice, and closely recapitulates the pathologic hallmarks of human PV. Administration of Aranesp 3 times over 7 days (qOD), for example, uniformly results in polycythemia (hct>65%), reticulocytosis (>25% reticulocytes) and splenomegaly (>600 mg, normal = 80 mg) in both B6 and Balb/c mice [17]. Thus, this Epo-stimulated model offers many of the major anatomic hallmarks of human PV without the JAK2^V617F^ mutation. We used this model of secondary polycythemia to investigate the role of the spleen in secondary, Epo-driven compared to cell autonomous, JAK2^V617F^-driven PV. Both B6 and Balb/c mice underwent SH or SPL operations, were allowed to recuperate for 2 weeks, and Aranesp was administered three times over 1 week. As shown in Fig. 5, both B6 and Balb/c mice readily develop polycythemia (67.5±1.7 and 66±0.8%) and splenomegaly (624±46 and 607±32 mg) after SH operation. Similarly, but in contrast to what is observed in JAK2^V617F^-driven PV, SPL mice also develop polycythemia to a level that is more than 80% of the increase seen from normal, and 90–92% of the total hematocrit seen in SH operated mice (Fig 5). Thus, a robust polycythemic phenotype develops in splenectomized mice in response to wild type (Epo→Epo receptor→Jak2) signaling. Furthermore, chronic injection of Aranesp over weeks, inducing persistent polycythemia and splenomegaly, fails to induce fibrosis in bone marrow or spleen (data not shown). Thus, the pathology of Epo-induced, secondary polycythemia contrasts sharply to JAK2^V617F^-driven PV, which requires an intact spleen and leads to fibrosis/osteosclerosis in hematopoietic tissues.

## Discussion

The observed differences between the role of the spleen in primary versus secondary polycythemia could be explained if 2 independent pathologic processes lead to elevation in hematocrit and bone marrow fibrosis, respectively. In the first, the spleen provides either a required factor or an anatomic niche for JAK2^V617F^-expressing cells, leading to erythrocytosis and increased hematocrit. These could be distinguished if it were possible to induce hematocrit elevation in splenic extracts versus splenic re-transplantation into splenectomized JAK2^V617F^-harboring mice. These mechanisms are not mutually exclusive, however, if the spleen elaborates a critical factor (Epo) in the PV context, for example, thus providing both ligand stimulation a pathogenic niche. In the second process, one or multiple of the amplified myeloid lineages (red cell, white cell, or platelet-forming) of JAK2^V617F^-expressing cells mediate fibrosis and osteosclerosis in the bone marrow, and this process occurs independently of elevations in hematocrit. If indeed essential thrombocythemia (ET), PV and primary myelofibrosis (PMF) represent a continuum of one disease that differ in JAK2^V617F^ gene dosage, then it will be critically important to know the gene dosage in patients prior to considering splenectomy [18]–[20]. This consideration would be important because SPL, although effective in lowering hematocrit, could also potentially alter disease progression the bone marrow.

An alternative possibility is that hematopoietic competition in the bone marrow space, in the absence of a spleen, prevents erythroid expansion specifically. That is, if JAK2^V617F^ –expressing erythroid, myeloid and megakaryocytic lineages in PV compete in the marrow space, then splenectomy could limit pathologic expansion. In contrast, pathologic stimulation of a single lineage of cells, via Epo for example, results in a homogeneously (erythroid) hyper cellular bone marrow that is sufficient to maintain elevated hematocrit. This model has been intensively tested in mice via high-dose, ligand-stimulation (G-CSF, Epo) competition experiments. In these experiments, co-administration of Epo and G-CSF to splenectomized mice only modestly reduces late stage granulocyte and erythrocyte lineages, respectively, and SCF prevents this mutual inhibition [21]. Thus, even maximal myeloid-expanding ligand stimulation has only a modest, and not preventative, effect on erythroid expansion even in the absence of a spleen. Further studies could test whether the arrest of erythroid expansion after SPL in PV is a result of competition between multiple hematopoietic lineages in a restricted space. Noting that the pathogenesis of myelofibrosis and erythrocytosis can be dissociated by removal of the spleen, one hypothesis is that the spleen provides a niche for specific populations of endogenous erythroid colony-forming cells (EECs). We were not able to grow EECs from V617F-transplanted Balb/c mice. Thus, additional studies might help to determine whether differences in EEC numbers or proliferation explain normal versus polycythemic phenotypes observed in SPL versus SH operated mice, respectively.

We recognize that secondary, Epo (Aranesp)-driven erythrocytosis represents a stronger phenotype than V617F-driven polycythemia due to the shorter time required for progression of disease (7 versus 14–28 days, respectively). Thus, a stronger erythropoietic drive has the potential to explain the presence (Epo) or absence (V617F) of polycythemia following SPL in these 2 models. Nonetheless, these 2 models achieve similar, maximal and stable levels of hematocrit (65%). Thus, if viewed in terms of maximal polycythemia observed, the effect of SPL has a marked, curative consequence in the mutant model, while only blunting the phenotype of Epo-induced polycythemia. Additional experiments, such as administering low-dose murine Epo chronically, or generating a mouse expressing low levels of Epo transgenically, would further address whether SPL has a similar effect of a similar time frame in secondary (Epo) versus mutant-driven (V617F) polycythemia.

Two strains of mice were used in these experiments in order to reduce the likelihood that any effects we measured were due to strain variations. Marked strain variations, similar to what has been reported, were observed with respect to bone marrow and spleen myelofibrosis [12]. For example, at 6 weeks of age the majority of Balb/c mice harbor myelofibrosis in the bone marrow, while none of the C57Bl6 (B6) mice show this abnormality (data not shown). This difference is observed despite using identical titers of V617F-containing virus, and despite similar levels of maximal hematocrit. Later, by approximately 12–14 weeks of age, more than 50% of the B6 mice show reticulin fibrosis in the bone marrow. In comparison to Balb/c mice, which show marked myelofibrosis at this stage, B6 mice show a more modest phenotype. This variable myelofibrotic phenotype resembles that seen in prior reports [12]. These observations agree with prior hypotheses, which suggest that unknown genetic modifiers result in a heterogeneous phenotype both between strains [12] and even within a single, transgenic line of mice [19].

The amount of EMH that takes place in the spleen is likely to be different in mice (high) than humans (modest). Nonetheless, our finding that SPL normalizes only the erythroid lineage in mice allows at least a theoretical extension to human PV. If, for example, the spleen is required for primary, JAK2^V617F^-driven PV, then all patients should have splenomegaly or at least EMH in the spleen. By clinical examination and ultrasound, the vast majority of patients with PV do indeed have evidence of splenomegaly [22]. The JAK2^V617F^ mutation can indeed be detected in the spleen as well as BM in patients with PV, and clonality suggests an origin in bone marrow [23]. Furthermore, noninvasive imaging with ^18^F-FLT PET shows evidence of EMH in all patients with PV, although patients with end-stage splenic fibrosis may not be expected to have this finding [24]. We are, furthermore, not aware of any cases of PV in patients who were asplenic prior to the onset of disease, although absence of this data cannot constitute proof. In order to clinically test the hypothesis that the spleen is required for PV, it will be necessary to first understand and control the abnormal rheology of blood, as well as the susceptibility to infections in PV patients undergoing splenectomy. If patients with PV can be effectively treated, spleen size is reduced, and this treatment lowers surgical risk, then the role of the spleen in human PV could be tested directly. We predict that, unlike other malignant conditions (CML) that are accompanied by splenomegaly, splenectomy in patients with PV will reduce hematocrit to normal levels. The consequences of residual JAK2^V617F^ burden in the bone marrow will be important to characterize further.

Despite the recent and remarkable discovery that the JAK2^V617F^ mutation plays a critically important role in the development of PV, much remains unknown about its pathogenesis. Others and we have designed specific inhibitors of JAK2 that may be useful therapies for PV, although their benefit over current treatment remains to be demonstrated [25]. Our data predict that spleen size itself will offer a highly sensitive, noninvasive measurement of disease, and that splenectomy will prevent the hematocrit-elevating consequences of JAK2^V617F^-associated disease. We propose that a cure for PV, however, will require reducing or ablating residual JAK2^V617F^ burden in the bone marrow. Interestingly, our finding that splenectomy in JAK2^V617F^-driven, PV improves erythroid neoplasia sharply contrasts sharply to studies which suggest that splenectomy may accelerate frank carcinogenesis [26]. Our observations are limited to the JAK2^V617F^ mutation, and cannot necessarily be extrapolated to the remaining 5% of mutations described in human PV [27]. Finally, these data show that the spleen-dependent JAK2^V617F^-driven polycythemia is pathologically independent of JAK2^V617F^-associated reticulin fibrosis and osteosclerosis in bone marrow. The role of the spleen in human PV, as well as other, clinically untested splenomegaly-associated diseases remains unknown.

## Supporting Information

Table S1 Hematopoietic and anatomic changes in mice who first underwent surgery (splenectomy or sham) and then secondarily received V617F-containing cells via bone marrow transplant. Surgery was performed, and then 2 weeks after surgery mice were transplanted with congenic bone marrow cells that were transduced with the V617F murine gene construct. After 7 weeks, analysis of cellularlity, V617F mutation positive (GFP+), erythroid (Ter119+) cells, complete blood count and organ size was performed. Vacant data in SPL columns are due to splenectomy performed at the start of treatment.
(0.14 MB PDF)

Table S2 Hematopoietic changes in mice who were transplanted with V617F-transduced bone marrow, and secondarily underwent splenectomy or sham operations. Mice were first irradiated, transplanted with congenic bone marrow cells harboring the V617F mutation, and then surgery was performed 4 weeks later (V617FBMT-Surgery). After 7 weeks, analysis of cellularlity, V617F mutation positive (GFP+), erythroid (Ter119+) cells, complete blood count was performed.
(0.13 MB PDF)
