# Supplementary material for: Splenectomy Normalizes Hematocrit in Murine Polycythemia Vera
Source: PLoS One. 2009 Sep 30;4(9):e7286. doi: 10.1371/journal.pone.0007286 (PMC2749451; doi:10.1371/journal.pone.0007286)
Supplement: Table S1 — Hematopoietic and anatomic changes in mice who first underwent surgery (splenectomy or sham) and then secondarily received V617F-containing cells via bone marrow transplant. Surgery was performed, and then 2 weeks after surgery mice were transplanted with congenic bone marrow cells that were transduced with the V617F murine gene construct. After 7 weeks, analysis of cellularlity, V617F mutation positive (GFP+), erythroid (Ter119+) cells, complete blood count and organ size was performed. Vacant data in SPL columns are due to splenectomy performed at the start of treatment. (0.14 MB PDF) [file pone.0007286.s001.pdf]

STab1

Surgery → V617F Transplant

	C57BL/6		BALB/c	
	SH	SPL	SH	SPL
**Bone Marrow**				
GFP+ cells ($x10^6$)	3.0 ± 1.3	3.0 ± 1.3	2.2 ± 0.9	1.0 ± 0.1**
GFP+ cells (%)	14.6 ± 6.2	16.2 ± 5.2	22.9 ± 9.0	26.7 ± 3.5
Ter119+ cells ($x10^6$)	12.5 ± 1.1	10.5 ± 1.2**	5.7 ± 1.0	1.7 ± 0.2**
Ter119+ cells (%)	60.7 ± 5.2	57.9 ± 6.5	58.9 ± 1.0	44.4 ± 4.7**
Cellularity ($x10^6$)	20.1 ± 4.6	20.3 ± 4.8	11.5 ± 2.7	6.5 ± 1.8**
**Peripheral Blood**				
WBC ($x10^3$/ul)	63.0 ± 35	23 ± 6**	153 ± 36	36 ± 7**
PLT ($x10^3$/ul)	2112 ± 751	919 ± 195**	1040 ± 282	948 ± 170
RBC ($x10^6$/ul)	13.9 ± 1.2	10.2 ± 1.9**	11.5 ± 0.9	7.2 ± 0.3**
RBC mass ($x10^9$)	29.1 ± 11.5	27.8 ± 8.2	15.6 ± 3.6	12.9 ± 1.8
**Spleen**				
Size (mg)	477 ± 119	NA	640 ± 82	NA
Total cells ($x10^6$)	772 ± 228		393 ± 100	
GFP+ cells ($x10^6$)	112 ± 73		32 ± 8	
GFP+ cells (%)	14.5 ± 9.4		10.1 ± 2.6	
Ter119+ cells ($x10^6$)	642 ± 29		236 ± 8	
Ter119+ cells (%)	83.0 ± 3.7		74.0 ± 2.4	
**Liver**				
Size (mg)	1229 ± 58	1131 ± 129	1247 ± 82	1217 ± 56

**, $P < 0.01$
