# Supplementary material for: Splenectomy Normalizes Hematocrit in Murine Polycythemia Vera
Source: PLoS One. 2009 Sep 30;4(9):e7286. doi: 10.1371/journal.pone.0007286 (PMC2749451; doi:10.1371/journal.pone.0007286)
Supplement: Table S2 — Hematopoietic changes in mice who were transplanted with V617F-transduced bone marrow, and secondarily underwent splenectomy or sham operations. Mice were first irradiated, transplanted with congenic bone marrow cells harboring the V617F mutation, and then surgery was performed 4 weeks later (V617FBMT-Surgery). After 7 weeks, analysis of cellularlity, V617F mutation positive (GFP+), erythroid (Ter119+) cells, complete blood count was performed. (0.13 MB PDF) [file pone.0007286.s002.pdf]

STab2

V617F BMT → Surgery

	C57BL/6		BALB/c	
	SH	SPL	SH	SPL
**Bone Marrow**				
Total cells ($x10^6$)	27.7 ± 5.6	15.5 ± 4.7**	8.7 ± 1.2	8.2 ± 1.1
Frequency live (%)	86.6 ± 2.2	79.5 ± 3.0**	78.4 ± 4.0	71.8 ± 6.3*
GFP^+ cells ($x10^6$)	9.7 ± 2.4	8.7 ± 2.1	4.1 ± 0.5	3.5 ± 0.7
GFP^+ cells (%)	34.0 ± 8.4	40.0 ± 9.9	47.9 ± 6.2	41.5 ± 8.1
**Peripheral Blood**				
WBC ($x10^3$/ul)	41.2 ± 6.6	47.1 ± 38.2	63.2 ± 25.9	25.9 ± 8.7*
PLT ($x10^3$/ul)	356 ± 111	528 ± 360	490 ± 124	619 ± 238
RBC ($x10^6$/ul)	14.2 ± 1.7	7.0 ± 0.8**	9.6 ± 1.3	6.4 ± 0.7**
RBC mass ($x10^9$)	17.3 ± 2.3	16.4 ± 5.8	NA	

*, P <0.05; **, P < 0.01
